# Peptide-Based Identification of *Phytophthora* Isolates and *Phytophthora* Detection in Planta

**DOI:** 10.3390/ijms21249463

**Published:** 2020-12-12

**Authors:** Miroslav Berka, Marie Greplová, Iñigo Saiz-Fernández, Jan Novák, Markéta Luklová, Pavla Zelená, Michal Tomšovský, Břetislav Brzobohatý, Martin Černý

**Affiliations:** 1 Phytophthora Research Centre, Department of Molecular Biology and Radiobiology, Faculty of AgriSciences, Mendel University in Brno, CZ-61300 Brno, Czech Republic; miroslavberka94@gmail.com (M.B.); saizfern@mendelu.cz (I.S.-F.); novakhonza@atlas.cz (J.N.); luklovam@gmail.com (M.L.); xzelena1@gmail.com (P.S.); brzoboha@ibp.cz (B.B.); 2 Potato Research Institute, Ltd., CZ-58001 Havlíčkův Brod, Czech Republic; greplova@vubhb.cz; 3 Phytophthora Research Centre, Department of Forest Protection and Wildlife Management, Faculty of Forestry and Wood Technology, Mendel University in Brno, CZ-61300 Brno, Czech Republic; michal.tomsovsky@mendelu.cz; 4 CEITEC–Central European Institute of Technology, Mendel University in Brno, CZ-61300 Brno, Czech Republic; 5 Institute of Biophysics of the Czech Academy of Sciences, CZ-61265 Brno, Czech Republic

**Keywords:** *Phytophthora*, proteomics, *P. palmivora*, *P. infestans*, *Solanum tuberosum*, *Hordeum vulgare*, leaf inoculation

## Abstract

*Phytophthora* is arguably one of the most damaging genera of plant pathogens. This pathogen is well suited to transmission via the international plant trade, and globalization has been promoting its spread since the 19th century. Early detection is essential for reducing its economic and ecological impact. Here, a shotgun proteomics approach was utilized for *Phytophthora* analysis. The collection of 37 *Phytophthora* isolates representing 12 different species was screened for species-specific peptide patterns. Next, *Phytophthora* proteins were detected in planta, employing model plants *Solanum tuberosum* and *Hordeum vulgare*. Although the evolutionarily conserved sequences represented more than 10% of the host proteome and limited the pathogen detection, the comparison between qPCR and protein data highlighted more than 300 protein markers, which correlated positively with the amount of *P. infestans* DNA. Finally, the analysis of *P. palmivora* response in barley revealed significant alterations in plant metabolism. These changes included enzymes of cell wall metabolism, ROS production, and proteins involved in trafficking. The observed root-specific attenuation in stress–response mechanisms, including the biosynthesis of jasmonates, ethylene and polyamines, and an accumulation of serotonin, provided the first insight into molecular mechanisms behind this particular biotic interaction.

## 1. Introduction

*Phytophthora* (Peronosporaceae, Peronosporales, Oomycota) is a globally distributed plant pathogen causing significant economic losses. The *Phytophthora* diversity and community composition in natural areas are influenced by many environmental factors, including precipitations and temperature [1]. Little is known about its presumptive coevolved roles in indigenous ecosystems, but introduced *Phytophthora* species are recognized as globally widespread primary parasites of a broad spectrum of plant species, including trees, shrubs, and crops [2]. Although some oomycetes have a biotrophic, saprophytic, or opportunistic necrotrophic lifestyle, most *Phytophthora* species are necrotrophic or hemibiotrophic plant pathogens [3,4]. Indeed, this genus is responsible for considerable economic, environmental, and cultural losses, such as late blight caused by solanaceous crops pathogen *P. infestans*, sudden oak death caused by *P. ramorum*, and the ink disease of the chestnut caused by *P. cinnamomi* [5,6,7].

*Phytophthora* species could cause both under-ground and above-ground plant damage, depending on their lifecycle. Infection by soilborne species results in wet rotting of the roots and collar, loss of fine roots, and bleeding bark cankers, while airborne species cause leaf necrosis, shoot blight, and fruit rot. The symptoms of the disease include reduced leaf size, chlorotic leaves, development of necrosis, and plant death [2,8]. Directly or indirectly, *Phytophthora* species are estimated to cause 66% of all fine root diseases and more than 90% of all collar rots of woody plants [9,10]. Thus, early detection and identification of *Phytophthor*a spread are of high importance to minimize its repercussion.

The means of controlling *Phytophthora* diseases are limited and require early detection of the pathogen. *Phytophthora* can be detected by plating diseased tissue or an infected bait onto selective agar and observing the morphological characters of the resulting mycelial growth [2,11]. However, this is a cumbersome time-demanding technique prone to false negatives and misidentifications due to morphological convergence of reproductive organs of various *Phytophthora* species [12]. This method is also limited by its low sensitivity and is very much dependent on the specific conditions of the given cultivation protocol [13]. Molecular methods based on immunodetection, such as enzyme-linked immunosorbent assays, are sensitive but not species specific and thus the most reliable approaches are based on qPCR, and PCR followed by DNA sequencing analysis for targeted quantitation and identification, respectively [8,9,10,14]. There is an ample amount of evidence that protein analysis can provide complementary data to gene-based approaches. Unlike ELISA, the present-day mass spectrometry (MS) proteomics techniques have shown high specificity and sensitivity and can be utilized for identification of microorganisms. For instance, it has been shown that culture classification using reference-based matrix assisted laser desorption/ionization MS (MALDI-MS) is comparable to that yielded by the 16S rRNA phylotype assignment up to the genus level [15]. However, the robust and rapid MALDI-MS has its limitations and more advanced techniques seem to be necessary for confident subgenus identifications. In contrast to the standard MALDI-MS whole-cell profiling, the tandem MS-based proteotyping and phylopeptidomics provide a larger number of recorded signals with better accuracy and precision, and the approach can manage complex microorganism mixtures [16,17].

Here, the possibilities of LC–MS-based *Phytophthora* mycelium identification were explored, and the peptide-based in planta detection was demonstrated.

## 2. Results

### 2.1. Peptide-Based Identification of Phytophthora Isolates

**Figure 1:**
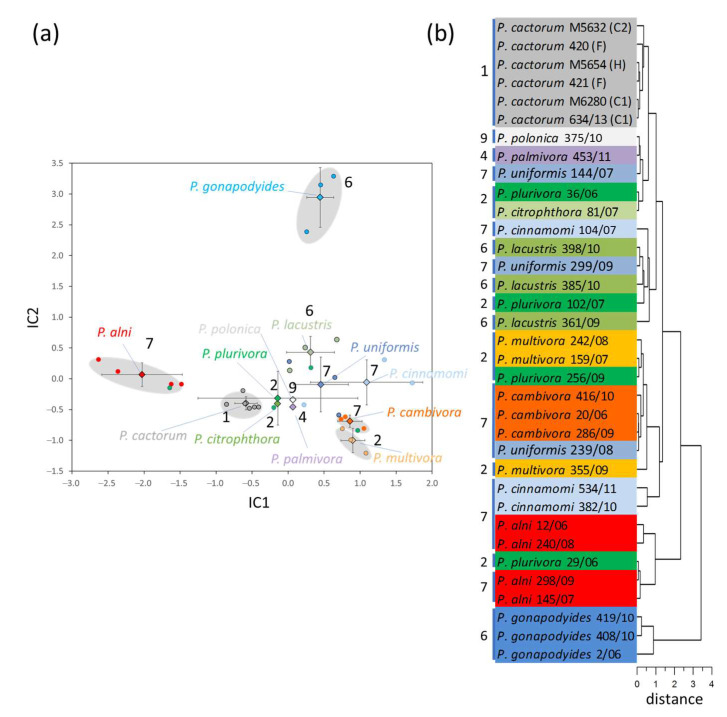
Shotgun proteomics-based clustering of *Phytophthora* isolates. (**a**) Independent component analysis of 1787 MS1 peak areas (intensity threshold—0.1% of the total ion chromatogram). Circles, diamonds, error bars and the gray shading represent mean profiles of two biologically distinct samples, means, standard deviation, and significant clustering of the species (*p* < 0.05; Kruskal–Wallis), respectively. Numbers indicate recognized *Phytophthora* clades, color corresponds to the individual species; (**b**) Clustering analysis based on IC1 and IC2 coordinates.

The mycelium proteome screening targeted 35 *Phytophthora* isolates selected from the Czech Collection of Phytopathogenic oomycetes (RILOG, The Silva Tarouca Research Institute for Landscape and Ornamental Gardening, public research institution). *Phytophthora* isolates representing six out of ten available *Phytophthora* clades were grown for up to two weeks at 22 °C on malt extract agar medium until the mycelium covered the whole Petri dish. Next, the mycelium was collected, and the total protein extracted employing a combination of phenol/acetone/trichloroacetic acid extraction and digested with trypsin. The resulting peptides were analyzed by nanoflow reverse-phase liquid chromatography-mass spectrometry (LC–MS). First, the raw spectra were processed to identify key features represented as unique combinations of m/z at the given retention time, and the independent component analysis was employed to visualize the resulting patterns (Figure 1a,b). The observed separation was statistically significant for *P. alni*, *P. gonapodyides*, *P. cambivora*, *P. multivora,* and *P. cactorum.* Isolates of *P. lacustris* and *P. cinnamomi* shared similar features with *P. uniformis*. It should be noted that *P. palmivora, P. polonica,* and *P. citrophthora* were underrepresented in the analysis, and it remains to be seen if the revealed proteome patterns are fully representative of these species.

### 2.2. The Most Abundant Species-Specific Peptides Predominantly Originated from Enzymes of Major Metabolic Pathways

**Figure 2:**
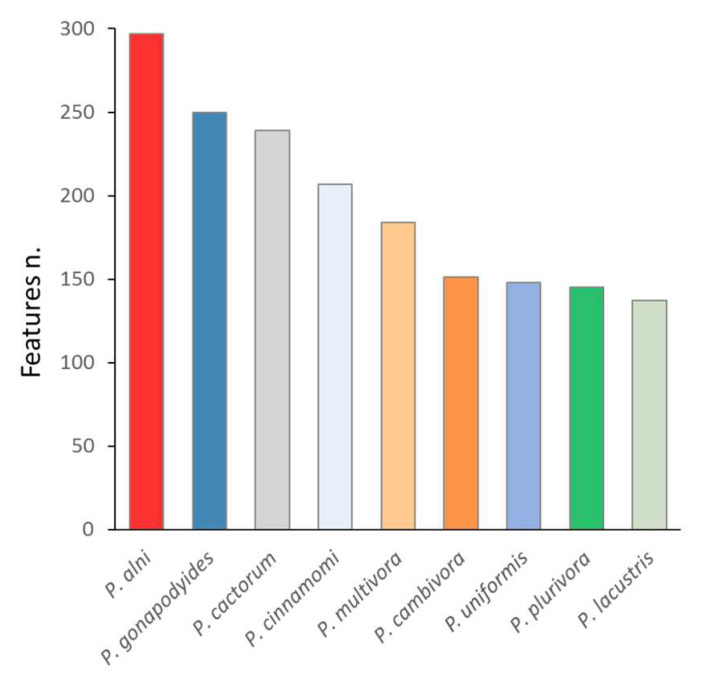
Species-specific patterns found in mycelium proteomes. Number of identified features showing statistically significant differences (Kruskal–Wallis test, *p* < 0.05) in contrast to all other analyzed species with at least three different isolates. Color corresponds to individual species (Figure 1). See Supplementary Table S2 for details.

The ICA separation clearly resolved at least five *Phytophthora* species. However, as illustrated in Figure 2, statistical analysis of species represented by more than one isolate showed that all contained species-specific patterns, including those that were not separated by IC1 or IC2. To provide an insight into the identity of peptides and proteins behind the observed species-specific patterns, the whole dataset was searched against all known *Phytophthora* proteins listed in the UniProt protein database. In total, more than 9500 putative peptide sequences were reported (Supplementary Table S1). Next, the most significant features per species were selected and matched against the list of identified peptides with the identical combination of m/z and retention time. In total, 107 out of 180 candidates were successfully matched and identified. Interestingly, the identifications were missing also for the isolates with the available reference genome, indicating the presence of unexpected modifications or spliced forms of *Phytophthora* genes. The detailed analysis revealed that the identified peptides predominantly originated from proteins of major metabolic pathways, including energy metabolism (22), carbohydrate-active enzymes (CAZymes; 15), ribosomal proteins (12), ROS metabolism (11), protein folding (8), or amino acid metabolism (6). At least three of the peptides, which displayed a species-specific pattern, originated from elicitors and secretory proteins, including a transglutaminase elicitor (*P. cactorum*), protoplast secreted protein (*P. gonapodyides*), and cysteine-rich secretory protein (*P. plurivora*). Surprisingly, only two proteins were represented by more than one peptide in the limited dataset, namely superoxide dismutase (*P. cambivora*) and ADP/ATP translocase (*P. multivora*).

### 2.3. Phytophthora Detection in Planta

*Phytophthora* mycelium on agar plates has distinct characteristics that are not found under natural conditions during its interaction with the host plants. Furthermore, the suitability of proteotyping *Phytophthora* isolates could be limited to specific cultivation conditions. To explore these differences in detail, we employed two model plants with a well-characterized proteome, *Hordeum vulgare* and *Solanum tuberosum*.

#### 2.3.1. Detection of *P. palmivora* Proteins in Infected Seedlings of *H. vulgare*

Recent studies found that *P. palmivora*, a well-known destructive pathogen of tropical plants, can infect the non-natural host barley (*Hordeum vulgare*) [18]. These findings presented an opportunity to employ a model crop plant and characterize the molecular changes induced by *P. palmivora*. Barley seeds were surface-sterilized, germinated on a filter paper and transferred onto a liquid medium containing *P. palmivora* as described in Materials and Methods. After 24 h, barley roots and shoots were separated, rapidly washed and flash-frozen in liquid nitrogen. Proteins were extracted and analyzed by LC–MS. Next, the measured peptide spectra were searched against the *P. palmivora* protein database. In total, 188 peptides matching *P. palmivora* sequences were identified, but most of these candidates were also in the mock-treated samples and are likely false positives. Only a portion of peptides identified in *P. palmivora*-treated roots was not detectable in the mock-treated samples (Supplementary Table S3), including secreted RxLR effector peptide protein (A0A2P4Y445), sulfate permease (A0A2P4YP98), and ATP-dependent RNA helicase (A0A2P4Y928). However, none of the putative *P. palmivora* proteins was present in the mycelium dataset.

#### 2.3.2. *P. palmivora* Altered Barley Metabolism and Stress Signaling

**Figure 3:**
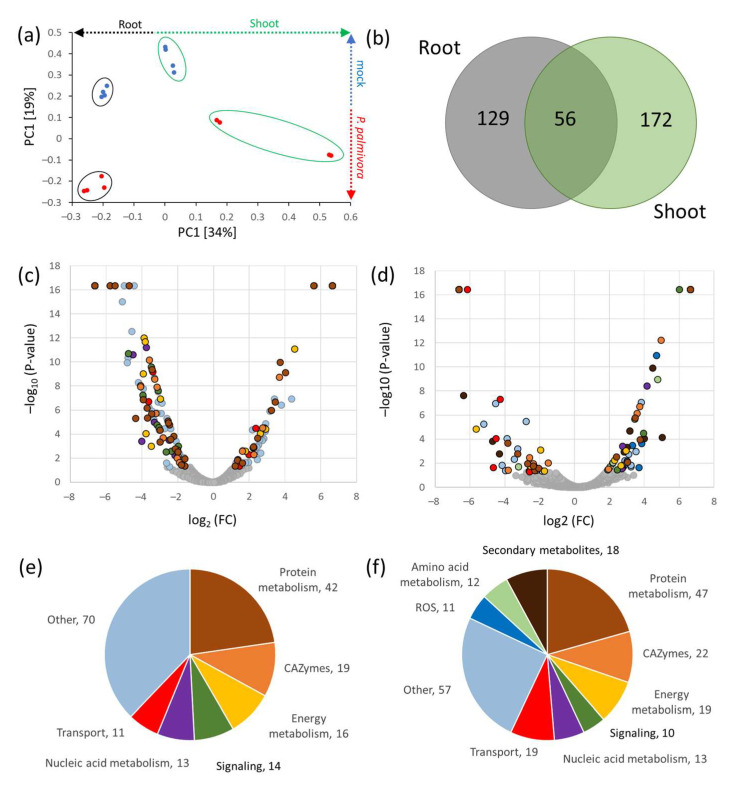
Barley proteome response to *P. palmivora*. (**a**) The proteome profile separation of *P. palmivora*-treated and mock-treated samples. Principal component analysis based on differentially abundant proteins in shoots and roots. Results of four biological replicates represent proteins with at least two matched unique peptides. Mock-treated plants (blue) and plants inoculated with *P. palmivora* (red) are indicated. Circles represent statistically significant separation (Kruskal–Wallis test, *p* < 0.05), black and green color corresponds to root and shoot tissue, respectively. (**b**) The overlap between differentially abundant proteins in shoots and roots (absolute fold change FC > 1.5; *p* < 0.05). (**c**–**f**) Volcano plot representation and functions of *P. palmivora* response proteins in barley roots (**c**,**d**) and shoots (**d**,**f**). Only categories represented by more than nine proteins are highlighted. The color of spots representing differentially abundant proteins in panels (**c**,**d**) corresponds to categories highlighted in (**e**,**f**).

The detection of *P. palmivora* proteins did not provide strong evidence for *Phytophthora* infection. However, the proteome analysis of barley showed a significant impact of *P. palmivora*, identifying 192 and 232 differentially abundant proteins in roots and shoots, respectively (Supplementary Table S4, Figure 3a–f; *p* < 0.05, absolute fold change FC > 1.5). As illustrated in Figure 3c–f, *P. palmivora* response proteins were functionally enriched in protein metabolism, CAZymes, energy metabolism, transport and nucleic acid metabolism. The detailed analysis of root proteome response highlighted the modulation of cell wall metabolism, including the depletion of pectin acetylesterase HORVU3Hr1G116470, two endoglucanases (HORVU1Hr1G062110, HORVU4Hr1G011160), and three proteins involved in lignin biosynthesis (HORVU2Hr1G086380, dirigent protein; HORVU2Hr1G086380, HORVU5Hr1G064260, shikimate O-hydroxycinnamoyltransferases), and the accumulation of an enzyme catalyzing biosynthesis of the primary cell wall polysaccharides (HORVU2Hr1G016840, 2-dehydro-3-deoxyphosphooctonate aldolase).

Interestingly, *P. palmivora* presence induced significant depletion of multiple proteins involved in the biosynthesis of signaling molecules and signaling, including ethylene biosynthetic enzyme 1-aminocyclopropane-1-carboxylate oxidase (HORVU1Hr1G020430), polyamine oxidase (HORVU7Hr1G118240), a subunit of COP9 signalosome complex (HORVU2Hr1G050230) with a putative role in jasmonate-mediated defense [19], and two isoforms of jasmonic acid biosynthetic enzyme 12-oxophytodienoate reductase (HORVU7Hr1G095960 and HORVU2Hr1G077220). In contrast, these jasmonic acid biosynthetic enzymes were accumulated in the shoot. A similar accumulation was found for several other defense-related proteins, including hypersensitive-induced response protein (HORVU7Hr1G017190) and two isoforms of cytochrome P450 (HORVU7Hr1G083670, HORVU2Hr1G00455), indicating stress response and ROS production in the shoot. The putative accumulation of reactive oxygen species in shoots likely resulted in the observed induction of ROS-repression mechanisms, namely monodehydroascorbate reductase (HORVU7Hr1G072240), glutathione peroxidase (HORVU7Hr1G030810), glutathione S-transferase (HORVU4Hr1G057740 and HORVU1Hr1G052470), or mitochondrial uncoupling protein HORVU4Hr1G027150 [20].

Differentially abundant proteins related to transport and trafficking were found in both tissues. The observed changes corresponded predominantly with protein depletion and seemed to be tissue-specific. There was only a limited set of proteins with a similar tissue-independent response, including accumulation of mitochondrial translocase subunit (HORVU7Hr1G054920) and depletion of tubulins (HORVU4Hr1G067370 and HORVU1Hr1G023030), calcium-transporting ATPase (HORVU1Hr1G007310), or putative transporter MlaD domain-containing protein HORVU5Hr1G010300. A contrasting response was found for Syntaxin-132 (HORVU6Hr1G095330), a protein with a putative role in membrane trafficking and root hair tip growth [21]. This protein was accumulated and depleted in shoots and roots, respectively. A similar profile was found for protein HORVU3Hr1G017970 with a putative role in root hair elongation (sharing 54% identity with *A. thaliana* protein GET4), but only its accumulation in shoots was statistically significant (*p* < 0.05) within the given set of biological replicates.

Finally, *P. palmivora* response in roots was analyzed by gas chromatography mass spectrometry (GC–MS), which provided quantitative data for 69 polar and semipolar metabolites. The results showed significant alterations in plant metabolome, including depletion in free amino acids and polyamines. For details, see Supplementary Table S5.

#### 2.3.3. Detection of *P. infestans* in Inoculated Detached Leaves of *Solanum tuberosum*

**Figure 4:**
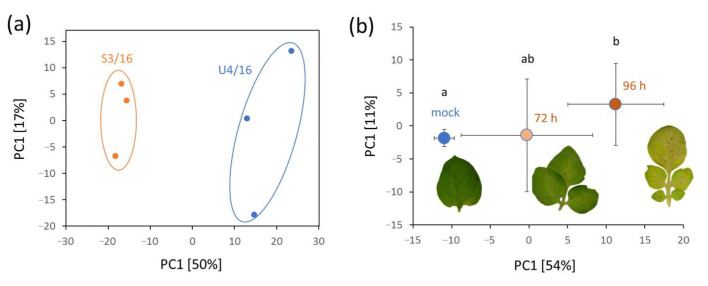
*Phytophthora infestans* peptide-based detection in mycelium and in planta. (**a**) Separation of proteome profiles of two independent isolates used for the evaluation *P. infestans* mycelium proteome. Based on relative abundances of most abundant proteins (786 identified proteins with at least two unique peptides); (**b**) representative images of plant material and separation of proteome profiles of *S. tuberosum* leaf 72 and 96 h after inoculating with *P. infestans.* Presented data visualized by PCA are means and standard deviation (*n* = 5), different letters indicate significant differences (Kruskal–Wallis test, *p* < 0.05). Based on relative abundances of most abundant proteins (408 identified proteins with at least two unique peptides).

The initial mycelia screening experiment targeted species associated with tree disease, and therefore *P. infestans* was not included in the dataset. Thus, mycelia of two different *P. infestans* isolates were sampled and analyzed, providing the reference dataset and quantitative data for more than 2000 proteins (Supplementary Table S6). Differences in the MS analysis did not allow the integration of these data into the original screening, but the separation of isolates was clearly visible, providing further evidence of the phylopeptidomics-based subspecies identification (Figure 4a). Next, the detached leaves of *S. tuberosum* (cv. Kerkovske rohlicky) were inoculated with a mixture of *P. infestans* isolates as described in Materials and Methods. Leaf samples were collected 72 and 96 h after the inoculation, and proteome profiles were compared to the mock-treated leaves (sampled after 96 h). More than 900 putative *P. infestans* proteins were detected in the plant tissue extracts, and proteome profiles showed a clear correlation with the infection progress, separating inoculated and mock-treated samples in the first PCA component (Figure 4b). 

**Figure 5:**
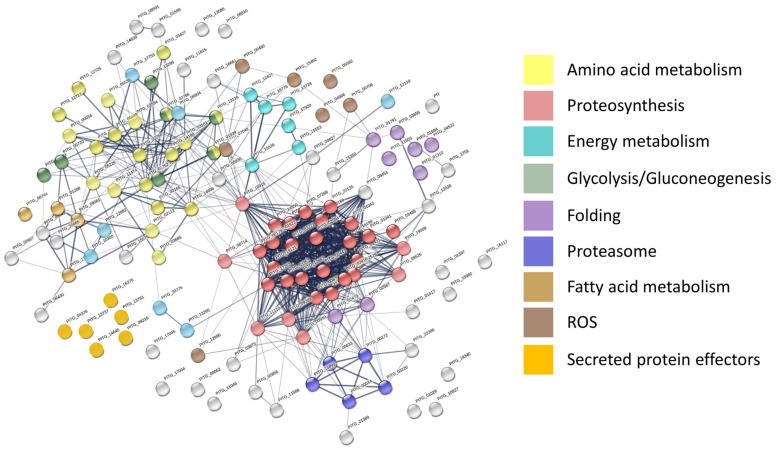
Interactions and functional clusters of high-confidence *P. infestans* proteins highlighted by STRING [22]. Color-coding of proteins is denoted by functional designation given by KEGG pathway enrichments, only nine most significant categories are highlighted.

The set of 146 high-confidence *P. infestans* proteins (containing at least two detected unique peptides and not identified in the mock-treated samples; Supplementary Tables S7 and S8) were analyzed in detail. Functional enrichment by String (Figure 5) showed that these were enzymes belonging to amino acid metabolism, ribosomal proteins, ROS metabolism enzymes, CAZymes, and components of proteosynthetic machinery and proteasome. Surprisingly, only six detected proteins were annotated as secreted effectors, namely protein elicitin (XP_002906009.1), two protoplast-secreted proteins (XP_002899456.1; XP_002899435.1), RxLR effector peptide protein (XP_002897665.1; XP_002903512.1), and a cysteine-rich secreted protein (XP_002898542.1). The comparison of the datasets revealed positive and negative correlations with pathogen exposure time for 37 and 2 proteins, respectively (Table S7). However, statistically significant differences between 72 and 96 h treatments (absolute fold change FC > 1.5; *p* < 0.05) were found only for five accumulated proteins (acetyl-coenzyme A synthase, XP_002909294.1; ATPase, XP_002896250.1; subunit of translation initiation factor 3, XP_002903574.1; and two proteins of unknown function, XP_002998370.1 and XP_002909937.1) and one depleted protein (60S ribosomal protein, XP_002909966.1).

#### 2.3.4. Detection of *P. infestans* in Field-Grown *Solanum tuberosum*

**Figure 6:**
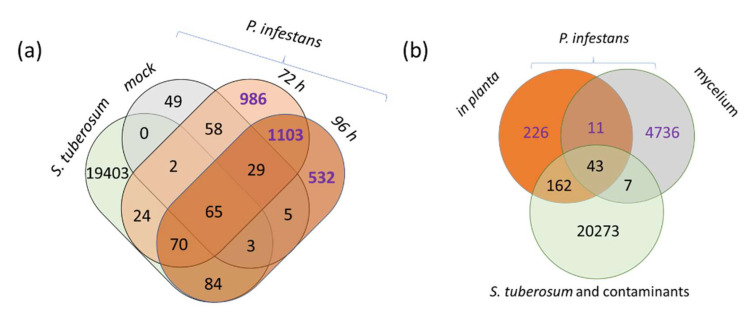
Venn diagram representation of identified peptides in inoculated detached leaves (**a**) and in leaves from the field experiment (**b**). Mock, 72 h and 96 h, in planta, mycelium—peptides found in the search against the *P. infestans* database in the respective sample group; *S. tuberosum—*peptides found in the search against the *S. tuberosum* database and common contaminants database. Purple color-coding indicates peptides unique for *P. infestans* proteome. See Supplementary Tables S7 and S8 for details.

For the second set of experiments aimed at the in planta detection of *P. infestans*, *Solanum tuberosum* (cv. Borek) was grown in an experimental field without any chemical control. Plants were regularly monitored, and at the first sight of *P. infestans* infection, 15 leaves of neighboring plants without any manifestation of visible symptoms at the time of sampling were collected and analyzed. The measured peptide spectra were searched against the *S. tuberosum*, *P. infestans* and common contaminants databases, and putative *P. infestans* proteins were identified. In total, 237 peptides (corresponding to at least 227 proteins) were detected after filtering out evolutionarily conserved peptide sequences shared between *S. tuberosum* and *P. infestans* (Figure 6b, Supplementary Table S9). The number of identified *P. infestans* proteins was lower than in the inoculated detached leaves, but results proved that this technique was successful in early *Phytophthora* detection.

#### 2.3.5. Validation of *P. infestans* Presence in Planta

**Figure 7:**
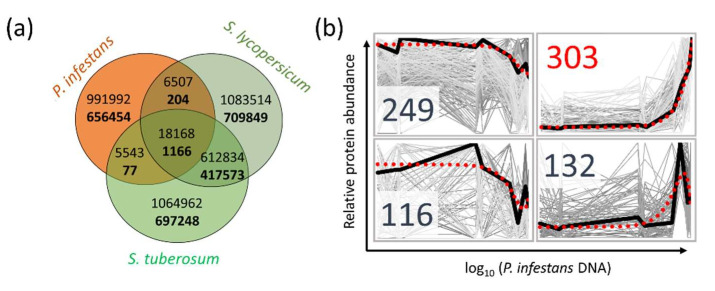
Elucidation of false-positive *Phytophthora* protein identifications. (**a**) Evolutionarily conserved peptide sequences in three model genomes digested by trypsin in silico. Numbers corresponding to low-stringency (maximum three miscleavages, 6–30 amino per peptide) and high-stringency criteria peptides (maximum one miscleavage, 8–25 amino acids per peptide) are represented in plain and bold font, respectively; (**b**) correlations between *P. infestans* DNA and protein relative abundances. Numbers indicate the size of the protein profile cluster. The mean profile and polynomial regression are represented by black and red curves, respectively. See Supplementary Tables S10 and S11 for details.

The high number of false-positive hits found in the mock-treated plants (Figure 6a,b) seemingly undermined the output of the peptide-based analysis. Two experiments were conducted to explain the observed discrepancies and provide further support for the peptide-based *Phytophthora* detection. First, in silico digests of *P. infestans*, *S. tuberosum,* and *S. lycopersicum* proteomes were compared and shared evolutionarily conserved sequences identified (Figure 7a). The results clearly illustrated an overlap, which was summarized in Supplementary Table S10. Next, the qPCR analysis was performed (Materials and Methods, Supplementary Table S14), and estimated *Phytophthora* protein and DNA amounts were compared. Protein profiles were clustered into four groups by the k-means clustering algorithm. As illustrated in Figure 7b, more than 300 out of 802 quantified proteins showed a strong correlation with the *P. infestans* DNA amount.

## 3. Discussion

### 3.1. Peptide-Based Analysis May Be the Future of Phytophthora Detection and Classification

Their considerable impact on the economy and environment is eliciting a growing interest in *Phytophthora* species, and the number of discovered *Phytophthora* species continues to grow [5,9,23,24,25]. The total number of formally named species in the genus has doubled in the last two decades [9,10,12] and sequencing-based identification employing only several genetic markers has become increasingly challenging. Protein-based *Phytophthora* detection has been predominantly limited to antibody-based methods. However, the ever-increasing sensitivity of the MS-based detection and promising development in nanopore sequencing technology [26,27] may soon promote protein-based detection to the method of choice for reliable *Phytophthora* identification.

The results demonstrated in this study showed that the feature-based comparison of trypsin-digested mycelium could resolve different species and differentiate isolates within the given species (Figure 1a, Figure 2, and Figure 4a). In contrast to a genome-based analysis, feature-based detection does not require any prior knowledge of (peptide) sequences and can be utilized to differentiate different isolates without the need for genome sequencing. The comparison employs hundreds of features and could be more reliable than standard approaches for phylogenetic classification. However, it should not be expected that the proteome-based clustering would perfectly match that of genome molecular phylogeny. First, the most abundant proteins are integral components of evolutionary conserved metabolic pathways and are shared between contrasting species. Further, it has been found that the proteome differences among and within species are lower than that expected from the corresponding gene expression [28]. Distant species may produce seemingly similar proteome patterns, and only detailed analysis will reveal contrasting features, such as observed here for the *Phytophthora* mycelium proteomes (Figure 1a,b). Unfortunately, this unsupervised detection is also prone to errors originating in proteome dynamics inherent to growth and development. For instance, it cannot be ruled out that the observed pattern of *P. plurivora* isolates is only a reflection of these dynamics.

### 3.2. Highly Abundant Mycelium Proteins Are Not the Best Targets for Phytophthora Detection in Planta

**Figure 8:**
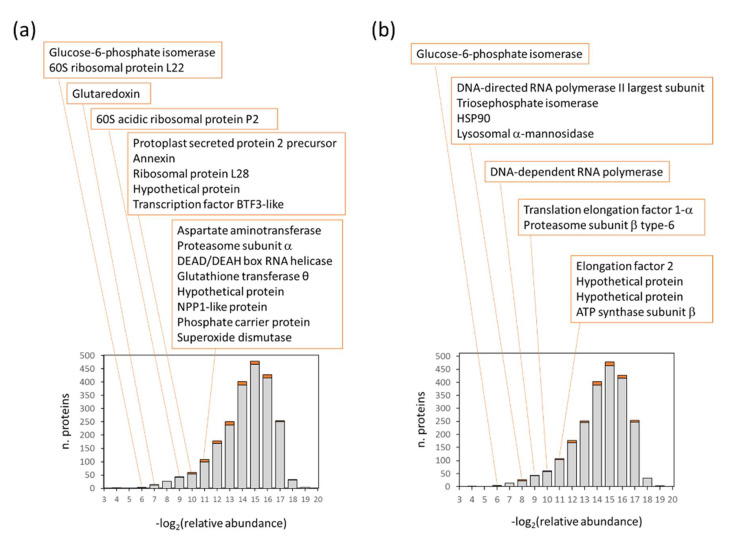
Relative abundance distribution of 2282 *P. infestans* proteins quantified in mycelium and highlighted proteins (orange) found in inoculated detached leaves (**a**) and in the field-grown plants (**b**). Proteins representing 1.6–0.05% of total protein abundance in mycelium are listed.

**Figure 9:**
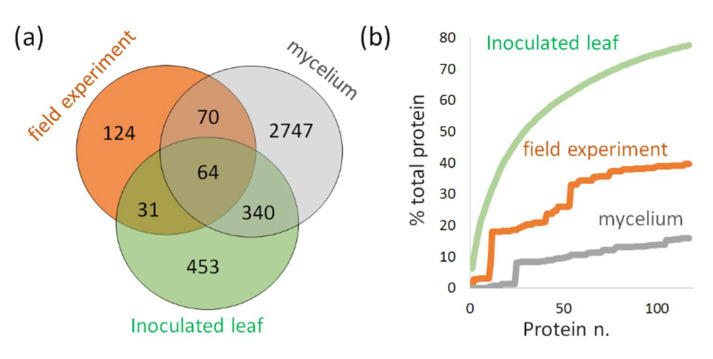
The comparison of *P. infestans* proteins found in all three datasets. (**a**) The overlap between identified *P. infestans* proteins and (**b**) the contribution of the most abundant *P. infestans* proteins found in the inoculated detached leaves (green) to the total *Phytophthora* protein content in leaves from the field experiment (orange) and mycelium (gray). See Supplementary Table S12 for details.

The comparison of mycelium and in planta datasets revealed only a limited overlap in identified peptides. However, the overlap in identified *Phytophthora-*specific proteins was higher, with 134 and 404 mycelium proteins detected in the field-grown plants and leaf-inoculation experiment, respectively (Figure 8a,b and Figure 9a, Supplementary Table S12). Most of these were not highly abundant in mycelium proteome (Figure 8a,b). It should be noted that the most abundant putative *Phytophthora* proteins were excluded from the list, namely histones H3 (XP_002906612.1) and H4 (XP_002906018.1) sharing nearly 100% identity with *S. tuberosum* orthologues.

The datasets for *P. infestans* indicated that the mycelium proteome is not the best reference for *Phytophthora* detection in planta. As illustrated in Figure 9b, 80% of the *P. infestans* protein abundance in the inoculation experiments corresponded to only 16% of total mycelium protein extract. This is lower than expected based on previous proteomics analyses [29], but similar results (11%) were found by comparing *P. cinnamomi* mycelium proteome with the recently published proteomics of *Castanea sativa* infection [30]. In contrast, the differences in *Phytophthora* protein abundances between field-grown plants and inoculated detached leaves were not that substantial. It seems that the leaf inoculation may represent a viable approach for generating suitable candidates for protein- and peptide-based *Phytophthora* detection in planta.

### 3.3. Evolutionary Conserved Protein Sequences Present an Obstacle for Unbiased Phytophthora Detection

The observed overlap between *Phytophthora* and its host proteome may limit the suitability of the peptide-based *Phytophthora* detection to model plants with a fully sequenced genome. To better understand these shared protein sequences, the whole-proteome digests were compared. Surprisingly, the number of matching *P. infestans* peptide sequences in *S. tuberosum* was low, representing only 0.1% of predicted high-stringency *S. tuberosum* peptides (Figure 7a). Similar results were found for *S. lycopersicum*, which shared 0.1% and 37.1% of peptides with *P. infestans* and *S. tuberosum*, respectively. However, the quantitative data showed that this minor contribution to the predicted peptidome originated from highly abundant evolutionary conserved proteins, including ATPase subunits, elongation factors, heat shock proteins, histones, ribosomal proteins, actins, tubulins, and primary metabolism enzymes (see Supplementary Table S10 for details). These proteins represented 13.6% of *S. tuberosum* leaf proteome, and as illustrated with histone H3 and H4, high sequence coverage may not be sufficient to determine the genome of origin. Plant orthologues of some of these proteins were previously implicated in biotic interactions [31,32,33] and it is thus tempting to speculate that this may in fact reflect a presence of pathogen proteome and not only the host’s response.

### 3.4. P. palmivora-Induced Alteration of Polyamine and Melatonin Biosynthetic Pathways May Be a Part of Host Defense Suppression Mechanism

There were no visible infection symptoms on barley seedlings after 24 h incubation with *P. palmivora*. However, the proteomic analysis clearly confirmed the host–pathogen interaction. To validate observed changes in barley *Phytophthora-*response proteins, root polar and semipolar metabolites were analyzed by GC–MS. The results showed that *P. palmivora* presence induced significant depletion (FC ≤ 2.0, *p* < 0.05) of free amino acids (Supplementary Table S5), which could at least partially correspond to the increasing demand for protein biosynthesis (ratio between estimated protein amount of *P. palmivora* accumulated vs. depleted proteins was 1.18). The depletion of leucine may also correlate with the depletion of isopropylmalate dehydrogenase (HORVU6Hr1G062440 and HORVU2Hr1G124400). Similarly, a decrease of polyamine oxidase could represent the feedback mechanism response to the depletion of polyamine putrescine. Lastly, serotonin was accumulated, and tryptamine was significantly depleted in response to *P. palmivora*. These are metabolites belonging to the melatonin biosynthetic pathway, and the observed changes indicated a putative increase in its production. Melatonin is a direct free radical scavenger and an inhibitor of plant ROS-induced cell death. Its putative accumulation coincides with an attenuated ROS response (compared to shoots) and a depletion of programmed cell death protein 4 (HORVU2Hr1G083610). It may be possible that the suppression of polyamines and the induction of melatonin are mechanisms by which *P. palmivora* disrupts the host defense mechanisms and facilitates infection.

### 3.5. Protein-Based Early Detection of Phytopthora in Planta

Hemibiotrophic oomycota, such as *P. infestans* and *P. palmivora*, employ a biphasic infection strategy, initially showing an asymptomatic biotrophic phase. During this stage, *Phytophthora* projects haustoria into plant cells that release virulence proteins known as effectors [34,35]. The experiment with barley presented here indicates that at least some of these secreted proteins can be detected in planta within 24 h of infection (Supplementary Table S3). Similarly, the comparison of *P. infestans* protein-based and qPCR-based detection showed over 300 protein markers that positively correlated with the detected amount of *Phytophthora* DNA. The sample from an asymptomatic leaf with the lowest amount of detected *P. infestans* DNA still contained more than 40 *P. infestans* proteins (Supplementary Table S11). However, the overlap with *P. infestans* proteins found in the asymptomatic leaves collected from the field experiment was lower, indicating a role of timing, environment, and systemic differences that cannot be captured in the detached leaves. On the other hand, these shared proteins such as RxLR effector (XP_002896928.1) and nucleoside diphosphate kinase (XP_002902842.1) may be suitable targets for the early *P. infestans* detection.

## 4. Materials and Methods

### 4.1. Isolates and Phytophthora Cultivation

*Phytophthora* isolates were obtained from the Czech Collection of Phytopathogenic oomycetes (RILOG, The Silva Tarouca Research Institute for Landscape and Ornamental Gardening, public research institution, Průhonice, Czech Republic) and Faculty of Agrobiology, Czech University of Life Sciences Prague (Dr. Jana Mazáková). The complete list of isolates is listed in Supplementary Table S13. Isolates were cultivated on malt extract agar (3% malt extract, 0.5% peptone, and 1.5% agar) at 22 °C for 7–14 days.

### 4.2. P. palmivora Response in Barley

*Hordeum vulgare* L. sensu lato (var. Sebastian) were cultivated as described before (Berka et al., 2020). In brief, seeds were surface sterilized, stratified for 48 h, transferred onto half-strength Murashige and Skoog medium and placed in a growth chamber providing 22 °C and 16/8 h light/dark cycles with 100 µmol m^−2^s^−1^ photon flux density during light periods. After 72 h, sets of 10 germinated seedlings were exposed to *P. palmivora* suspension or mock. *P. palmivora* suspension was prepared as follows. Medium suitable for elicitin production (2% glucose, 0.1% asparagine, 0.05% KH_2_PO_4_, 0.05% yeast extract, 0.025% MgSO_4_.7H_2_O, and 1 × 10^−4^% thiamine) was inoculated with *P. palmivora* mycelium or kept intact (mock). After seven days at 22 °C and 60 RPM, suspensions were diluted 1:1 with a half-strength Murashige and Skoog medium and used for barley infection. After 24 h, root and shoot tissue was separated and snap-frozen in liquid nitrogen. The whole experiment was done in four biological replicates with at least ten seedlings per replicate.

### 4.3. S. tuberosum Leaf Inoculation

*Solanum tuberosum* (cv. Kerkovske rohlicky) plants were cultivated for six weeks on modified Schenk and Hildebrandt medium (supplemented with 1.5 × 10^−4^ *w*/*v* Alar 85, 3 × 10^−4^ *w*/*v* AgNO_3_, 1.5% sucrose, and 0.3% Gelrit) at 22 °C and 16/8 h light/dark cycles with 60 µmol m^−2^s^−1^ photon flux density during light periods. Fully developed leaves were detached, rapidly submerged in water suspension of *P. infestans* (5 × 10^3^ spores mL^−1^) and transferred onto a pad of damp cotton wool in a Petri dish. Leaf samples were collected 72 and 96 h after inoculation in five biological replicates and were snap-frozen in liquid nitrogen.

### 4.4. S. tuberosum Field Experiment

*Solanum tuberosum* (cv. Borek) were sawn and cultivated at the experimental field under natural conditions. After 15 weeks, the first symptoms of *P. infestans* were detected. Six neighboring symptomless plants were selected and three randomly selected healthy leaves per plant were collected and snap-frozen in liquid nitrogen.

### 4.5. Protein Extraction and LC-MS Analysis

Total protein extracts were prepared as previously described [36] employing a combination of phenol/acetone/TCA extraction. Digested peptides were measured using a 15 cm C18 Zorbax column (Agilent, Santa Clara, CA, USA), a Dionex Ultimate 3000 RSLC nano-UPLC system (Thermo Fisher, Waltham, MA, USA), a qTOF maXis Impact mass spectrometer (Bruker, Bremen, Germany), or the Orbitrap Fusion Lumos Tribrid Mass Spectrometer (Thermo Fisher) as described previously [37,38].

### 4.6. Proteomics Data Processing

The acquired spectra were recalibrated and analyzed by Profile Analysis 2.0 (Bruker). The resulting matrix of intensities and m/z was processed by ICA (www.rapidminer.com; [39]). For peptide and protein identification, the acquired spectra were searched against the reference *Phytophthora* (https://www.uniprot.org/) and plant proteomes (https://plants.ensembl.org) databases by Proteome Discoverer 2.2–2.4 (Thermo Fisher), employing Sequest HT, MS Amanda 2.0 [40] or MSFragger [41] with the following parameters:

- qTOF data (Supplementary Tables S1 and S2)—mass tolerance MS1 35 ppm, MS2 0.05 Da; enzyme—trypsin, maximum two missed cleavage sites; and modifications—up to three dynamic modifications including Met oxidation and Asn/Gln deamidation.
- Lumos data (in planta experiments, *P. infestans*)—mass tolerance MS1 5 ppm, MS2 0.02 Da; enzyme—trypsin, max two missed cleavage sites; modifications—up to three dynamic modifications including Met oxidation and Asn/Gln deamidation; Met-loss (protein N-terminus); Cys carbamidomethylation; and the MS Fragger algorithm was employed exclusively with Lumos data and the default settings for mass-tolerant search (MS1 tolerance 500 Da). The quantitative differences were determined by employing precursor ion quantification by Profile Analysis 2.0 and the spectral counting method (qTOF), and by Minora, followed by normalization and a background-based *t*-test for peptide- and protein-based quantitation. For selected candidate proteins, the corresponding peptide peak areas were manually evaluated in Skyline [42]. The mass spectrometry proteomics data have been deposited to the ProteomeXchange Consortium via the PRIDE [43] partner repository with the dataset identifier PXD022569.

### 4.7. Detection of P. infestans by qPCR

DNA was extracted with the mixture of phenol:chloroform:isoamyl alcohol (25:24:1). The aqueous phase was precipitated with ammonium acetate and isopropyl alcohol, the resulting pellet was washed with 70% (*v*/*v*) ethanol, dried on vacuum evaporator, and resuspended in 50 μL of water. *P. infestans* was detected according to Llorente et al. [44], by amplifying *PiO8*, a highly repetitive sequence from its genome, and normalization to *S. tuberosum* gene *Ef—1α* (PGSC0003DMG400023270). qPCR was performed using a Light Cycler 480 Instrument II (Roche, Basel, Switzerland) with the primers and amplification conditions listed in Supplementary Table S14. Three independent biological replicates and four technical replicates were included for each PCR amplification. The relative amount of DNA was calculated using serial dilutions of a DNA stock solution.

### 4.8. GC–MS Metabolomics

Polar metabolites were extracted and analyzed as described previously [45], employing a Q Exactive GC Orbitrap GC–tandem mass spectrometer and Trace 1300 Gas chromatograph (Thermo Fisher). Samples were analyzed in three biological replicates.

## 5. Conclusions

The methods of unbiased *Phytophthora* detection have been predominantly focused on genomics approaches. This work provides evidence that the protein/peptide-based analysis of mycelium might be successfully utilized for differentiating *Phytophthora* species and isolates. The method sensitivity for early detection in planta may be comparable to that of qPCR, but extensive homology in abundant evolutionary conserved sequences is presently limiting its application.

## Supplementary Materials

Supplementary materials can be found at https://www.mdpi.com/1422-0067/21/24/9463/s1. Table S1: Mycelium proteome of *Phytophthora* species. Supplementary tables to Figure 1; Table S2: The most significant species-specific features. Supplementary tables to Figure 2; Tables S3–S5: *P. palmivora* proteins detected in planta, barley metabolites and proteins identified in response to *P. palmivora*. Supplementary tables to Figure 3; Tables S6–S9: Mycelium proteome of *P. infestans* and *P. infestans* proteins identified in planta. Supplementary tables to Figure 4, 6–8; Tables S10 and S11: Results of in silico proteome digest and protein correlation with *P. infestans* DNA in planta. Supplementary tables to Figure 7; Table S12: Identified *P. infestans* proteins in all experiments. Supplementary table to Figure 9; Tables S13 and S14: Complete list of isolates and parameters of qPCR analysis. Supplementary tables to Materials and Methods.
